# The Inter-Relation between Leptin Receptor (*Q223R*) Gene Polymorphism and the Risk of Egyptian Patients with HCC 

**DOI:** 10.31557/APJCP.2020.21.12.3557

**Published:** 2020-12

**Authors:** Hala A. Karam, Sahar S. Bessa, Ehab M. M. Ali, Thoria Diab, Tarek M. Mohamed

**Affiliations:** 1 *Department of Chemistry, Division of Biochemistry, Faculty of Science, Tanta University, Tanta, Egypt. *; 2 *Department of Internal Medicine, Faculty of Medicine, Tanta University, Tanta, Egypt. *; 3 *Department of Biochemistry, Faculty of Sciences, King Abdulaziz University, Jeddah, Saudi Arabia. *; 4 *Department of Chemistry, Faculty of Science, Tanta University, Tanta, Egypt. *

**Keywords:** Leptin, leptin receptor, liver cirrhosis, NASH, HCV, HCC

## Abstract

**Background:**

The relationship of leptin (LEP) and polymorphism of leptin receptor (LEPR) were studied in patients with hepatocellular carcinoma (HCC) and compared with those with liver cirrhosis to find out the extent of the risk of LEPR on patients with HCC.

**Methods:**

Serum LEP level and *LEPR Q223R *gene polymorphism were determined in 300 patients with liver disease categorized equally into five groups’ healthy volunteers, patients with hepatitis C (HCV), patients with non-alcoholic steatohepatitis (NASH), liver cirrhosis and HCC. *LEPR* gene was amplified by polymerase chain reaction (PCR) then digested by the MSP1 restriction enzyme.

**Results:**

The isolated 212 bp of LEPR was sequenced. The serum LEP level was reduced in patients with cirrhotic and HCC. Serum LEP level had negatively correlated with both tumor grade and size in HCC patients. The data obtained from restriction fragment length polymorphismPCR and sequencing revealed the existence of a novel synonymous Q223R single nucleotide polymorphism (SNP) in exon 223 of *LEPR* gene (1137101). LEPR Gln223Arg, GG and GA genotypes were found in all studied groups. LEPR Gln223Arg, AA genotype was found in NASH, HCC, and control. LEPR Gln223Arg GA genotype is associated with some patients with HCC.

**Conclusion:**

GA genotype of *LEPR Gln223Arg* may be regarded as a probable genetic risk factor for Egyptian patients with HCC.

## Introduction

Liver cancer is one of the most dangerous types of cancer and occurs as a result of chronic liver disease and hepatic fibrosis resulting from hepatitis B and C and chronic liver diseases (El-Zayadi et al., 2005). HCC is the third leading cause of death from cancer and the ﬁfth most frequent malignancy globally. One million new HCC cases were estimated and approximetly the same number of deaths occur every year (Siegel et al., 2017). 

Epidemiological studies have found that people are more likely to be obese, might be exposed to risk of liver cancer. However, the pathogenesis of this association is not fully understood. It seems that there is a relationship between adipocytokines, such as LEP, and HCC but the molecular mechanisms have not been clarified yet (Caldwell et al., 2004). Many studies have demonstrated genetic variants to be associated with HCC susceptibility (Ma et al., 2018, Ye et al., 2018). 

LEP is a peptide hormone secreted from adipose tissues. LEP binds with many LEPRs which several isoforms. LEP controls various metabolic processes as well as satiety and controls body temperature. Also, LEP stimulates pathophysiological conditions, such as hematopoiesis and polycystic ovarian syndrome. Despite the relationship of LEP to people with obesity, the changes of LEP and LEPRs might be related to the risk of many types of cancer (Wang et al., 2004, Swellam and Hamdy, 2012, El-Daly et al., 2020).

Leptin receptors belong to the cytokine receptors family, which is a type of transmembrane protein. When LEP binds to LEPRs, it stimulates the angiogenesis of cancerous cells. The gene expression of *LEPRs* increases in malignant liver, breast, and lung cancer cells. Moreover, the relationship between the increased gene expression of *LEPRs* and the death of patients with HCC was reported (Li et al., 2012, Tang et al., 2020, El-Hussiny et al., 2017). LEP binding with LEPRs stimulates signal pathways that activate protein kinases and correlates to proliferative of cancer cells (Zhang et al., 2018).

The most common in genetic changes are the various forms of individual change of nucleotides, identifies single nucleotide polymorphisms (SNPs) in the human genome, coding, and non-coding regions. There are many nucleotide changes in LEPR, including the LEPR Lys109Arg (rs1137100), Gln223Arg or Q223R (rs1137101) Lys656Asn (rs8179183), Ala976Asp, Pro1019Pro, Ser492Thr and Ser343Ser. Among them, Lys109Arg (rs1137100) and Gln223Arg (rs1137101) polymorphisms are most commonly studied. The two nonsynonymous SNPs may modify the function and the structure of LEPR protein (Matsuoka et al., 1997, Qiu et al., 2017).

The SNP of *LEPR *gene; Gln223Arg is characterized by G transition instead of A in exon 6 and codon number 223 that replace acidic amino acid glutamine (Gln or Q) to basic amino acid arginine (Arg or R) (CAG to CGG) in in the extracellular domain of LEPR. The positive charges of LEPR Gln223Arg change the capability of LEP and LEPR and folding of transducing signal ability of LEPR (Yiannakouris et al., 2001). 

There is a correlation between the SNPs of the LEPR and the likelihood of a tumor occurring in humans. LEP binds to mutant LEPR that altered Gln to Arg leads to changes in LEP signaling in the genetic expressions of the key cancer genes and consequently might exposed to the possibility of the breast, lung, oral, and colon cancer. It is possible that there is a relationship between the SNPs of LEPR and HCC (Snoussi et al., 2006, Li et al., 2012).

This study aimed to assess between the level of the LEP hormone and LEPR Gln223Arg in Egyptian patients with liver cirrhosis that leads to HCC. The relationship between the clinical-pathological characteristics of patients with HCC and the genetic polymorphism of *LEPR* had been clarified.

## Materials and Methods

*Human Subjects*

In this study, patients and healthy subjects were divided into five groups. Group I: control group; which included 50 healthy individuals. Group II: HCV group included 50 patients. Group III: NASH group included 50 patients. Group IV: The cirrhotic group included 50 patients. Group V: HCC group included 100 patients. The characteristic of each group was illustrated in Table (1). Blood samples were drawn from the patients according to the established ethical rules and approval of the ethical committee at the Faculty of Medicine of Tanta University was obtained (Code No. 31152/10/16). This work was done from December 2016 to June 2018. Patients were diagnosed with biochemical analyzes and imaging (ultrasonography and computed tomography) to confirm that patients with chronic liver disease. Some liver biopsy was also taken from some patients to find liver cancer. Patients with other diseases associated with chronic liver disease were excluded such as renal, cardiac and autoimmune. Patients with liver cancer who had undergone a previous hepatectomy, trans-arterial chemoembolization or radiofrequency ablation or any other suspected malignancies were excluded from this study.

*Methods*

Blood samples were collected from each individual and plasma and serum were separated to determine prothrombin time, liver function tests , serum AFP level by using the Human ELISA Kit, catalogue number (EF6011) , anti HCV antibodies, by using Rue de l’Industrie 8, B-1400 Nivelles, Belgium Anti-HCV ELISA. HCV RNA, hepatitis B surface antigen (HBsAg) by Rue de l’Industrie 8, B-1400 Nivelles, Belgium HBsAg ELISA kit, and hepatitis B core antibodies (HBcAb) by using ELISA technique, Monolisa Anti-HBc Plus-Bio-Rad. The serum leptin level was determined by using the DRG,ELISA leptin kit, catalogue number (EIA- 2395). Whole blood was collected in EDTA tube for determination *LEPR* gene polymorphism.

DNA was extracted from the blood using Qiagen QIA amp DNA Blood Mini Kit, catalogue number (D3006). The concentration of the DNA level was measured by Nanodrop. DNA purity was confirmed using 1% agarose gel electrophoresis.

The method of restriction fragment length polymorphism (RFLP) was carried out after the polymerase chain reaction used. The forward and reverse primer of the LEPR Gln223Arg region were selected from the data genomic bank base were Forward: 5’ACC TCT GGT TCC CCA AAA AG-3’, Reserve: 5’ TCA TCA TTT TAG TGC ATA ACT TAC CC-3’. Taq polymerase, dNTP and RNase free water were purchased from FastGene Taq Ready Mix Kit, catalogue number (LS26). 400 ng/μl of extracted genomic DNA was prepared. 8μl genomic DNA was added to 25μl mixture of Taq polymerase and dNTP. 2μl of each primer (0.8 μM) was added. The final volume was completed to 50μl using RNase free water. The thermal cycler model was TC-3000, USA. It was automated at 35 amplification cycles. The amplification conditions of each cycle was first adjusted to at 95°C for 3 min to initial denaturation of DNA and followed by DNA denaturation to at 95°C for 30 sec, annealing at 56°C for 30 sec and extension at 72°C for 1 min, followed by final extension at 72°C for 7 min.

The PCR product (212bp) was purified by using the isolate PCR purification kit (QIAamp Mini kit, catalogue no. 51104). The amplified DNA fragment of the *LEPR *gene was digested with restriction enzyme MSP1 (Thermo Scientific, USA) which produced two fragments 161bp and 51bp in homozygous allele which were visualized in 2% agarose gel.

The purified products of PCR were performed to MacroGen Company (South Korea) to be sequenced in both directions using ABI 3730XLDNA sequencer (Applied Biosystem, USA). The sequences were analyzed using the Chromas Lite 2.1 program (http://technelysium. com.au/?page_id=13). The identity of the sequenced PCR product, alignments, annotations, and assembly of the sequences were examined using Blast search against the GenBank database of Homo sapiens LEPR (http://blast. ncbi.nlm.nih.gov/Blast.cgi). 

*Statistical Analysis*

Graph Pad Prism, version 6.0 (Graph Pad Software Inc., California USA) was used to analyze and draw the figures of data. Most of the data were expressed as mean ± SD. Student’s t-test and one-way ANOVA and Chi-square test were applied to compare between the groups and the association between genotypes in the different classified groups respectively. The correlation between 2 parameters was determined by Pearson correlation coefficient (r) was applied to correlate between two parameters. The odds ratio (OR) and 95% confidence interval (CI) were used to estimate the relative risk and strength of association for their various genotypes. A p-value of <0.05 was considered a signiﬁcant value.

## Results

The clinical and biochemical characteristics of patients with liver disease and HCC were demonstrated in Table 1. The Serum alpha-fetoprotein was increased 99, 79, 90 and 39 folds in HCC patients as compared to healthy subjects, and patients with HCV, NASH and liver cirrhosis (Table 1). 

The serum leptin level was elevated in patients with NASH, than control (p < 0.001). It was lower in patients with HCC and cirrhotic patients than control (p<0.05, p< 0.01 respectively). It was significantly decreased in patients with HCC as compared to patients with NASH (p < 0.001). No significant difference was observed between patients with HCV and the control (Table1). 

**Figure 1:**
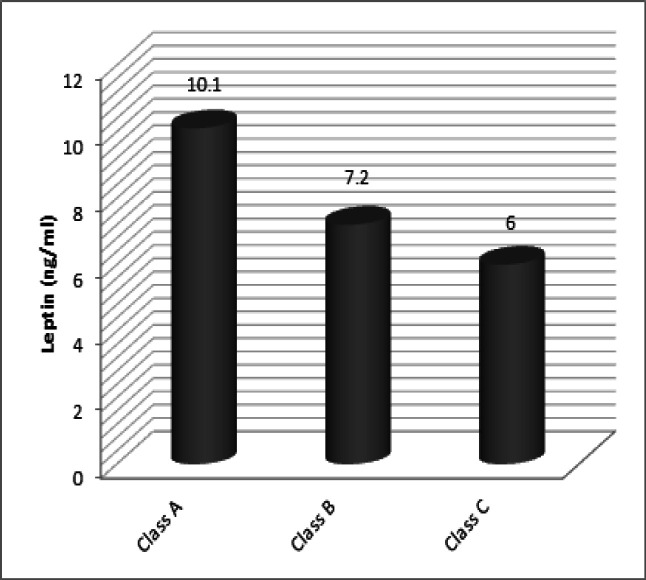
Serum Leptin Level Categorized According to Child-Pugh Classification in Patients with Liver Diseases and HCC

Serum LEP level in patients with liver diseases and HCC was categorized in the Child-Pugh classification (Fig 1). The mean values of serum LEP level were 10.1, 7.2 and 6 ng/ml in class A, B and C of patients with liver diseases or HCC respectively according to the Child-Pugh classification. Serum LEP level was reduced in patients with liver diseases or HCC in class C than that in patients in class A. 

**Figure 2:**
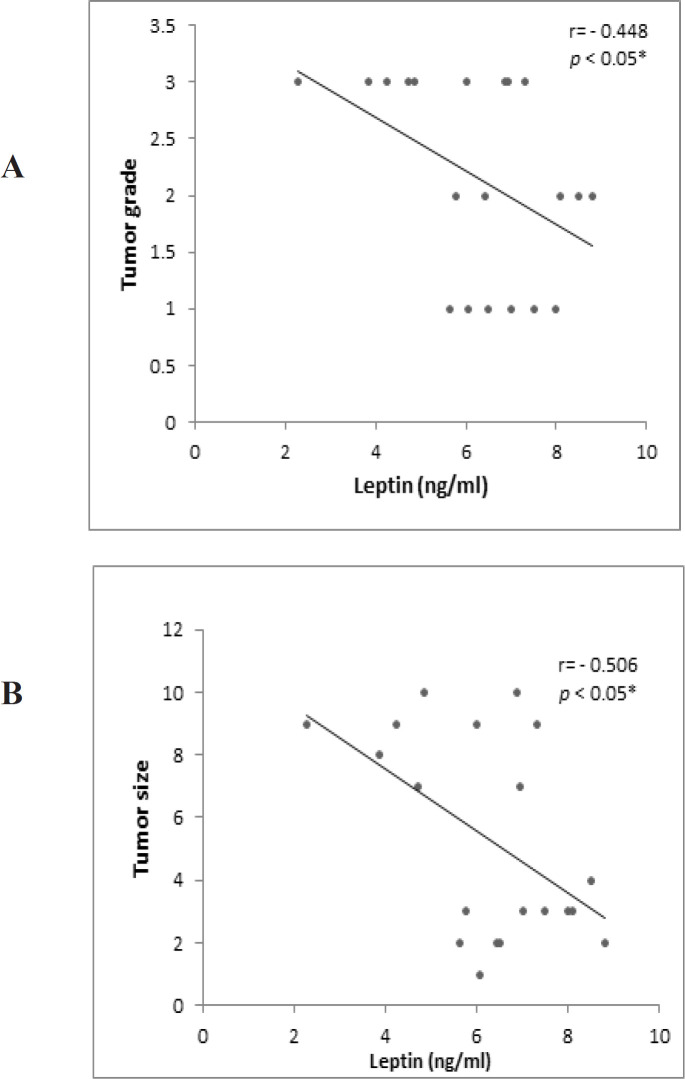
Correlation between Serum Leptin Level and Tumor Grade (A), and Tumor Size (B) in Patients with HCC

Serum LEP level and LEPR Gln223Arg genotypes in patients with HCC were categorized according to clinicopathological features of patients with HCC (Table 2). The serum LEP level was significantly decreased in patients with tumor size > 5 (p<0.05) than that in tumor size < 5. The correlation between serum LEP level and both tumor size and tumor grade in patients with HCC was slightly negative correlated (Figure 2). 

**Figure 3:**
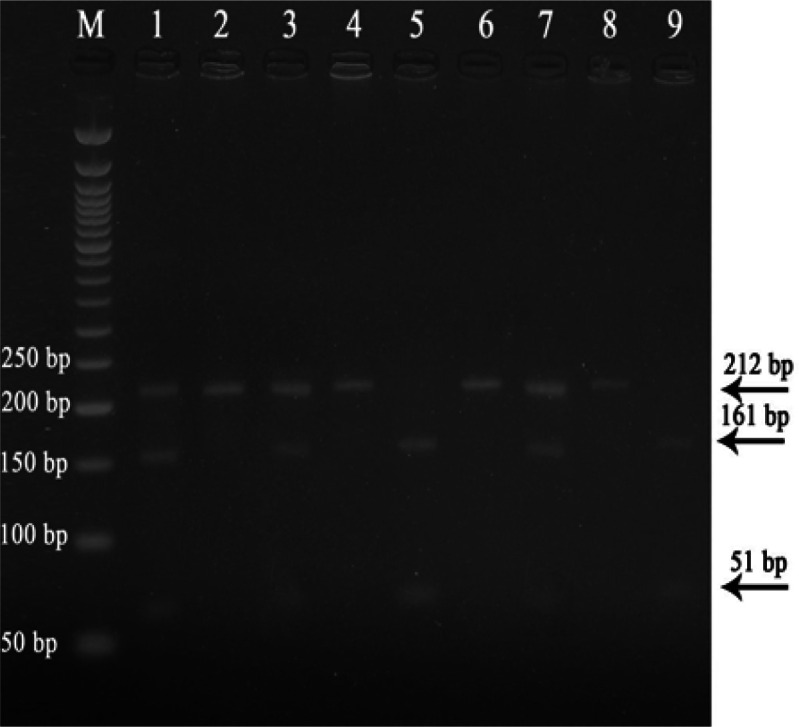
Representative Agarose Gel Electrophoresis for the Digestion of PCR-RFLP Products of LEPR Single Nucleotide Polymorphism where: Lane M represents DNA ladder; Lanes 2,4,6,8 represent wild type (GG); Lanes 5,9 represent homozygous mutant (AA); Lanes 1,3,7 represent heterozygous type (GA)

PCR RFLP method was used to detect genotypes for LEPR SNP (rs1137101). The genotypes of rs1137101 were shown in Figure 3. Table 2 demonstrated that there was no relationship between the clincopathological features including the tumor size, tumor grade, TNM stage and Child Pugh class and LEPR Gln223Arg genotypes.

The genotype LEPR Gln223Arg distribution was GG = 25 / 50 (50%), GA= 20/50 (40%) and AA = 5/50 (50%) in healthy subjects. The genotype distribution was GG = 15 / 50 (30%) and GA= 35/50 (70%) in patients with HCV. The genotype distribution was GG = 25 / 50 (50%), GA= 20/50 (40%) and AA = 5/10 (10%) in patients with NASH. The genotype distribution was GG = 10/ 50 (20%) and GA= 40/50 (80%) in patients with liver cirrhosis. While, in HCC patients, the genotype distribution was GG = 60 / 20 (60%), GA= 35/10 (35%) and AA = 5/10 (5%). The value of Chi-Square *χ*^2^ = 77.41, p < 0.0001 (Table 3).

Serum LEP level in the studied subjects classified according to LEPR Gln223Arg genotypes. NO significant difference was observed in serum LEP levels according to LEPR genotypes Figure 5. Table 2 shows the association between LEPR Gln223Arg genotypes in relation to clinicopathological features of HCC patients that were no relation between the clinicopathological features such as the tumor size, tumor grade, TNM stage and Child Pugh class and LEPR Gln223Arg genotypes (all p>0.05). 

Table 4 showed that LEPR Gln223Arg (GA) genotypes are associated with the risk of HCC (p<0.001) but LEPR Gln223Arg (AA) genotypes are not associated with the risk of HCC.

**Table 1:** The Clinical and the Biochemical Characteristics of Patients with Liver Disease and HCC

Characteristic	Controls (n=50)	HCV (n=50)	NASH (n=50)	Liver Cirrhosis (n=50)	HCC (n=100)
Age (Years)	55.0±1.5	56.0±3.3	57.3±3.5	55.3±4.7	59.8±5.1
Gender					
Male (n)	20	30	15	20	75
Female (n)	30	20	35	30	25
BMI (Kg/m^2^)	22.0±2.8	25.5±3.0	25.7±2.7	25.3±2.9	25.32±3.7
Child-Pugh					
Class A	-	40	45	15	30
Class B	-	8	5	30	40
Class C	-	2	-	5	30
HBsAg	-	-	-	5	15
HBcAb	-	-	-	10	20
Anti-HCV	-	50	-	45	85
ALT (U/L), mean±SD	15.7 ± 4.1	29.3±5.3	32.1±7.4	47.5±18.2	50.7±17.03
AST (U/L), mean±SD	19.6 ± 3.3	30.5 ± 6.5	45.8± 6.4	75.7 ± 8.2	102.5± 14.6
ALP (U/L), mean±SD	62.5 ± 8.6	65.7±8.9	97.7±10.3	99.7±22.5	108.4±20.18
Total bilirubin (mg/dl), mean±SD	0.7 ± 0.13	0.6 ± 0.14	0.86± 0.56	1.5 ± 1.2	2.6 ± 1.8
Albumin (g/dl)	4.6 ± 0.3	4.2±0.3	3.2±0.6	2.9±0.8	2.4±0.6
Prothrombin activity (%), mean±SD	96.2 ± 5.9	92.7±5.6	84.8±2.8	61.6±9.8	54.6±8.0
Serum AFP (ng/ml), mean±SD	3.6 ± 0.25	4.5 ± 1.19	4.0 ± 0.77	9.1 ± 2.55	357.52±262.9
Leptin (ng/ml), mean±SD	9.5 ± 3.4	8.0 ± 1.2	14..2 ± 4.4	5.4 ± 0.9	6.3 ± 1.7

**Table 2:** Serum Leptin Level and LEPR Gln223Arg Genotypes in Relation to Clinicopathological Features of Patients with HCC

	Serum Leptin (ng/ml)	P value	LEPR Gln223Arg (No. of Genotypes)			P-value
			GG	GA	AA	
Age						
<50	4.4±1.2	<0.05*	5	5	0	<0.05*
>50	6.6±0.37		55	30	5	
Sex						
Male	6.4±0.39	N.S	45	25	5	N.S
Female	6.2±1.1		15	10	0	
AFP(ng/ml)						
<200	6.6±0.46	N.S	25	30	5	<0.05
>200	5.9±0.69		35	5	0	
Tumor size		<0.05				0.05
<5 cm	7.1±0.34		25	30	5	
>5 cm	5.5±0.66		35	5	0	
TNM						
I, II	6.6±0.51	N.S	5	20	25	N.S
III	5.9±0.59		40	10	0	
Tumor grade						
Well, diff.	7.0±0.4	N.S	35	15	0	N.S
Moderate – poor diff.	6.0±0.53		25	20	5	
Child-Pugh Class						0.05
A	6.9±0.39	N.S	30	5	0	
B+C	5.5±0.7		30	30	5	

**Table 3:** Distribution of Genotypes of LEPR Gln223Arg among Patient with Liver Diseases and HCC

	Group 1		Group 2		Group 3		Group 4		Group 1	
	Control (n=50)		HCV (n=50)		NASH (n=50)		Liver Cirrhosis (n=50)		HCC (n=100)	
	n	%	n	%	n	%	n	%	n	%
Gln223Arg										
GG	25	50	15	30	25	50	10	20	60	60
GA	20	40	35	70	20	40	40	80	35	35
AA	5	10	-		5	10	-		5	5
x^2^			77.41							
*P*-value	< 0.0001									

**Table 4:** The Association between LEPR Gln223Arg Genotypes and the Risk of HCC

	Subjects without HCC n=200	Subjects with HCC n=100	OR (95% Cl)	P-value
Age (Years)				
<50	105 (52.5)%	10 (10%)	1.0	0.109
>50	95 (47.5)%	90 (90%)	2.0 (0.85 4.8)	
BMI (Kg/m^2^)				
<25	50 (25%)	15 (15%)	1.0	0.079
>25	150 (75%)	85 (85%)	1.8 (0.92 – 3.8)	
LEPR Gln223Arg				
GG	75 (37.5%)	60 (60%)	1.0 (reference)	
GA	115 (57.5%)	35 (35%)	3.7 (1.31 – 10.7)	0.001*
AA	10 (5%)	5 (5%)	0.61 (0.16 – 2.27)	0.468

**Figure 4:**
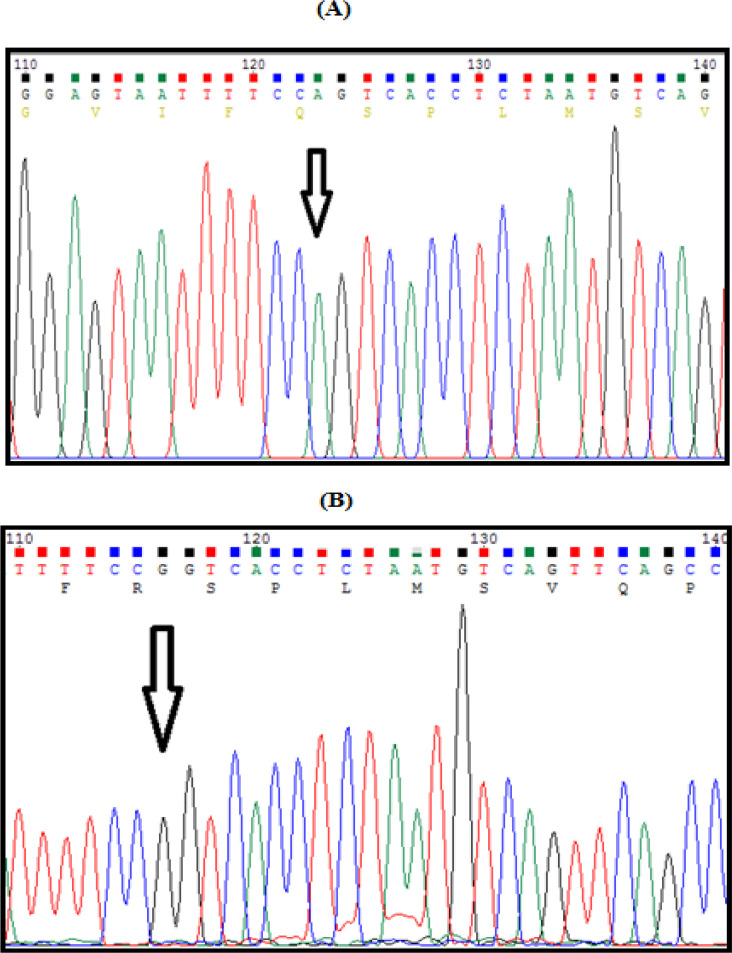
Electropherogram of LEPR Gln223Arg. (A), A allele - amino acid is Glutamine (Q); (B), G allele – amino acid is Arginine (R)

**Figure 5:**
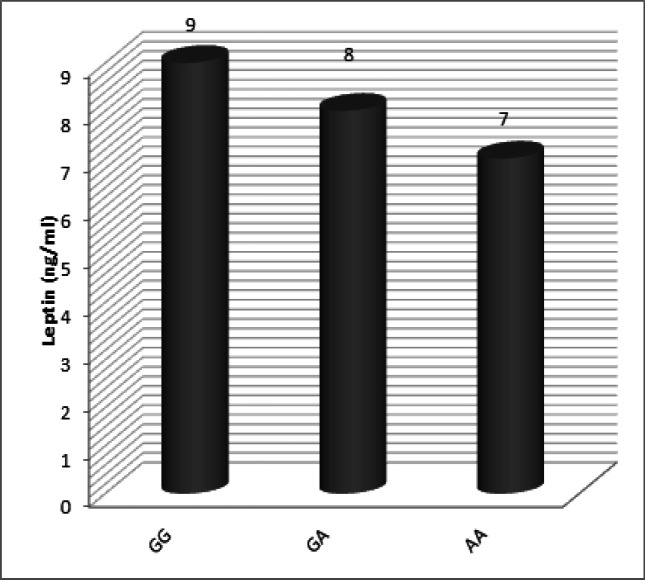
Serum Leptin Level in the Studied Subjects Categorized According to LEPR Gln223Arg Genotypes

## Discussion

*Liver cancer is the third most common cause of tumor worldwide*

In Egypt, HCC is the first among cancers in males (33.6%), and the 2nd in females after breast cancer (13.5%), with HCV genotype 4 is the most prevalent underlying cause (Siegel et al., 2017, Ibrahim et al., 2014) .The percentage of liver cancer in Egyptians increases significantly. Over the decades, we have found an increase in the incidence of HCC with an increased prevalence of HCV and less prevalence of Virus B as risk factors (El-Zayadi et al., 2005). This study was conducted to assess between LEPR Gln223Arg and serum LEP level in Egyptian patients with liver cirrhosis that leads to HCC and to clarify its relationship with the clinical-pathological characteristics of patients with HCC.

In this study, serum LEP level was elevated in NASH patients and reduced in HCC and cirrhotic patients as compared to control but no significant differences were found in the HCV group as compared to control. Similar results were obtained by Uygun, Kadayifci(Uygun et al., 2000), who reported that serum LEP level was significantly increased in NASH patients as compared to controls. The serum LEP level in chronic viral hepatitis patients did not differ from that in healthy subjects. There is a correlation between obesity and a high level of serum leptin. 

Also, Ataseven et al. (2006) found that leptin decreased in HCC and cirrhotic patients. The circulating leptin levels are increased in alcoholic cirrhotic patients (Ockenga et al., 2000). Others have found that serum leptin levels are low in posthepatitic cirrhotic patients (Onodera et al., 2001, Bolukbas et al., 2004). In addition, the nutritional status of cirrhotic patients represents a large range, from normal to severe malnutrition, related to the severity of the disease (Bolukbas et al., 2004).

These findings are supported by Greco et al., (1998) reported that the degree of malnutrition and fat mass in posthepatitic cirrhosis is due to the decrease in circulating levels of leptin. Thus, hyperleptinemia could be a consequence of fat mass reduction and malnutrition in HBV or HDV- related cirrhosis.

On the contrary, McCullough et al., (1998) observed that liver cirrhotic patients had increased in serum LEP levels. Also, Wang and Lin, (2003) reported that cirrhotic patients with and without HCC had increased in serum LEP levels, but did not appear to be associated with the development of HCC in cirrhotic patients. Moreover, Uygun et al., (2000) confirmed that high LEP levels in liver cirrhosis may be associated with hepatic failure rather than to host immune response or chronic inﬂammation to hepatic cell injury. Notably, serum LEP levels are similar in chronic viral hepatitis patients and healthy subjects point out that high serum LEP levels in NASH do not occur simply from liver damage. 

The LEP level was not related to high HCC development risk. This result was similar to previous studies on breast, endometrial, prostate cancers, and suggested a higher role of LEP in animal cancer models than in human carcinogenesis (Petridou et al., 2002). In the present study, it was found that serum leptin level was slightly reduced in patients with liver diseases or HCC in Child- C than that in patients in Child-A. Serum LEP level in patients with HCC was negatively correlated with both tumor size and grade.

This is supported by Greco et al., (2000) showed that patients with post-hepatitic cirrhosis Child-C had signiﬁcantly decreased LEP levels as compared to Child-A patients and healthy subjects. However, Campillo et al., (2001) found that alcoholic cirrhotic patients Child-C had increased leptin levels than those with Child-A and -B. Ockenga et al., (2000) who reported that LEP levels did not differ in post-hepatitic cirrhotic patients Child-A, B and C. Also, Henriksen et al., (1999) found no correlation between the severity of liver diseases (characterized by either Child-Pugh class or presence/absence of ascites) and leptin in alcoholic cirrhotic patients.

It is worth noting that LEPR is found in many tissues, such as the liver. LEPR was high level in HCC tissues and there was a signiﬁcant correlation between the expression of LEPR and micro-vessel density in tumor tissue. In addition, LEPR expression was a signiﬁcant determinant of HCC prognosis (Linjawi and Hussain, 2012).

Therefore, gene mutation may be the key to the occurrence of HCC (genetic susceptibility). This prompts us to check the association between LEPR Gln223Arg and the occurrence of HCC. In this study, LEPR Gln223Arg, GG genotypes were found in patients with HCV (30%), NASH (50%), cirrhotic (20%), HCC (60%) patients and control (50%). LEPR Gln223Arg GA genotype was represented in 40% of controls, 70% of chronic hepatitis C patients, 40% of NASH patients, 80% of cirrhotic patients and 35% of HCC patients. LEPR Gln223Arg, AA genotype was found in patients with (10%) NASH, HCC (5 %) and control (10%). This is compatible with Li, et al., (2012) who found that Lys109Arg and Gln223Arg polymorphisms were associated with the HCC risk but did not relate to the clinicopathologic features.

In the current study, when LEPR rs1137101 GA and AA genotypes were compared with the wild type (GG) of LEPR Gln223Arg, it was found that a significant difference in LEPR rs1137101 GA genotype between subjects with and without HCC and no difference was reported in the LEPR rs1137101 AA genotype between subjects with and without HCC. This means that the LEPR Gln223Arg GA genotype significantly affected the HCC risk. 

To date, only three studies studied the association of the rs1137101 and rs1137100 polymorphisms in LEPR with the risk of HCC, and results of these studies were opposite (Dai et al., 2010, Li et al., 2012, Zhang et al., 2018) . Therefore, the association of this polymorphism with HCC risk may be unclear. A study was made in 2010, by Dai et al., (2010) reported the association between HCC risk and LEPR polymorphisms. They involved 102 healthy controls and 82 patients with HCC, and found a positive association between HCC risk and the LEPR rs1137101 polymorphism. Li et al.(2012) reported in a 2012 study of 551 cancer-free controls and 417 conﬁrmed cases that the* LEPR* gene 1137101 polymorphisms were positively related with HCC. So, these results were agreement with our study.

Another study was made by Zhang et al., (2018) included 923 control cases and 584 HCC cases, found no association between the *LEPR* gene 1137101 polymorphisms and HCC risk. This result was in disagreement with our ﬁndings.

Notably, other SNPs of *LEPR* and *LEP* gene polymorphism were related to the HCC risk such as LEP rs7799039 A>G is a promoter SNP, which might affect the expression of LEP and LEPR rs6588147 G>A polymorphism decreased the HCC risk. In the Chinese Han population, the susceptibility of HCC related to LEP rs7799039 A>G polymorphism. Recently, a meta-analysis found that a decreased risk of cancer may associate with LEP rs7799039 A>G polymorphism (Zhang et al., 2018). In addition, this relationship between the cancer risk and LEP rs7799039 A>G polymorphism was reported in other meta-analyses (Liu et al., 2015).

Also, the genetic risk factor for HCC in an Eastern Chinese Han population is LEP rs2167270 G>A polymorphism which may affect the expression of the *LEP* gene. A functional study found that serum LEP levels in the LEP rs2167270 GG genotype were lower than in individuals with LEP rs2167270 GA genotype (Marcello et al., 2015, Zhang et al., 2018). 

Interestingly, Marcello et al., (2015) reported that high leptin levels had been related to the progression and incidence of several tumors such as HCC. In addition, LEP signaling pathways, such as signal transducer and activator of transcription 3 (STAT3) and PI3K/Akt, affect the growth, invasion, angiogenesis, and metastasis of HCC cells. Moreover, Watanabe et al., (2011) found that there is a correlation between high LEP levels and susceptibility to HCC recurrence in stage I/II case curatively treated by surgical resection or RFA. High serum LEP level may be a biomarker for predicting the HCC recurrence in high-risk patients. Furthermore, reported that the angiogenic response in HCC biopsy specimens was reduced by anti- LEP antibodies. Low serum LEP levels are also related to the prevention of obesity-related liver tumorigenesis in obese mice models.

In conclusion, in the present study, we found the serum LEP level was reduced in patients with cirrhotic and HCC. Serum LEP level had negatively correlated with both tumor grade and tumor size in HCC patients.. GA genotype of LEPR Gln223Arg may be regarded as a probable genetic risk factor for Egyptian patients with HCC. 
